# Características sociodemográficas e indicadores de preferencia de la población demandante de Manifestaciones Anticipadas de Voluntad

**DOI:** 10.1016/j.aprim.2025.103431

**Published:** 2026-01-14

**Authors:** María Victoria Castañeyra Góngora, Xenia Cabrera Rodríguez

**Affiliations:** Hospital Universitario Doctor José Molina Orosa, Las Palmas, España

**Keywords:** Voluntad en vida, Satisfacción del paciente, Derecho a morir, Eutanasia, Advance Directive, Patient Satisfaction, Right to Die, Euthanasia

## Abstract

**Objetivo:**

Analizar las características sociodemográficas, las preferencias y el grado de satisfacción de quienes registraron su Manifestación Anticipada de Voluntad (MAV) en Lanzarote.

**Diseño:**

Estudio observacional, descriptivo, prospectivo y transversal

**Lugar:**

Oficina de Manifestaciones Anticipadas de Voluntad de Lanzarote, Hospital Universitario Doctor José Molina Orosa (Servicio Canario de la Salud).

**Participantes:**

Personas mayores de 18 años que acudieron a registrar su MAV entre enero y diciembre de 2024.

**Medidas principales:**

Variables sociodemográficas, motivaciones para formalizar la MAV, preferencias consignadas y nivel de satisfacción.

**Resultados:**

Se analizaron 527 usuarios (67,2% mujeres; edad media 59,5 ± 14,3 años). La mayoría tenía estudios secundarios o superiores (65,7%) y no profesaba religión (78,7%). La motivación principal fue ejercer la autonomía personal (38,5%), seguida del deseo de no ser una carga (33,2%). Un 73,2% expresó su voluntad de donar órganos y un 86,7% se acogió a la eutanasia en demencia moderada-grave. Se hallaron asociaciones significativas entre el nivel educativo y la solicitud de eutanasia (χ^2^[6] = 39,27; *p* < 0,001), entre el motivo de realización y el sexo (χ^2^[4] = 11,70; *p* = 0,020) y la religión (χ^2^[20] = 463,49; *p* < 0,001). El 99,1% manifestó satisfacción con la atención recibida.

**Conclusiones:**

El perfil predominante es el de una mujer española, de edad media-avanzada y con estudios superiores. La autonomía al final de la vida constituye la principal motivación, con diferencias según sexo y creencias religiosas. Los resultados subrayan la necesidad de reforzar la formación profesional y las estrategias de comunicación sobre la MAV.

## Abstract (en)

**Objective:**

To analyze the sociodemographic characteristics, preferences, and satisfaction levels of individuals who registered their Advance Directive (Manifestación Anticipada de Voluntad, MAV) in Lanzarote.

**Design:**

Observational, descriptive, prospective, and cross-sectional study.Setting. Advance Directive Office of Lanzarote, University Hospital Doctor José Molina Orosa (Canary Health Service).

**Participants:**

Individuals aged 18 years or older who registered their MAV between January and December 2024.

**Main measurements:**

Sociodemographic variables, motivations for completing the MAV, stated preferences, and level of satisfaction.

**Results:**

A total of 527 users were analyzed (67.2% women; mean age 59.5 ± 14.3 years). Most had secondary or higher education (65.7%) and reported no religious affiliation (78.7%). The main motivation was the desire to exercise personal autonomy (38.5%), followed by the wish not to be a burden (33.2%). Organ donation was indicated by 73.2% of participants, and 86.7% accepted euthanasia in the context of moderate dementia. Significant associations were found between educational level and acceptance of euthanasia (χ^2^(6) = 39.27; p< 0.001), and between the motivation for completing the MAV and both sex (χ^2^(4) = 11.70; p = 0.020) and religion (χ^2^(20) = 463.49; p< 0.001). Overall, 99.1% reported being satisfied with the care received.

**Conclusions:**

The predominant profile was that of a Spanish woman, middle-aged or older, with higher education. Autonomy at the end of life emerged as the main motivation, with differences observed according to sex and religious beliefs. The findings highlight the need to strengthen professional training and communication strategies to promote the use of Advance Directives.

## Introducción

Las Manifestaciones Anticipadas de Voluntad (MAV), también denominadas documento de instrucciones previas o voluntades anticipadas, constituyen un instrumento legal mediante el cual una persona mayor de edad expresa los cuidados o tratamientos médicos que desea recibir o rechazar en el futuro. Este documento adquiere especial relevancia cuando el paciente pierde la capacidad de expresar su consentimiento, garantizando así el respeto a su autonomía y dignidad en la toma de decisiones sanitarias^1^.

La aprobación de la Ley Orgánica de Regulación de la Eutanasia (LORE) 3/2021, de 24 de marzo^2^, ha conferido una nueva dimensión a este registro, al reconocerlo como el medio legalmente válido para solicitar la prestación de eutanasia en caso de pérdida de capacidad. Esta normativa ha supuesto un avance en la legislación española al consolidar el derecho a una muerte digna y reforzar la importancia del documento MAV. Aunque su registro es posible en Canarias desde 2006, sigue siendo poco conocido entre la población y los profesionales sanitarios^3^.

El ejercicio de este derecho permite anticipar decisiones y evitar intervenciones consideradas desproporcionadas o contrarias a la voluntad personal, como la prolongación artificial de la vida en enfermedades incurables, demencias avanzadas o estados vegetativos. Su utilización proporciona tranquilidad tanto a las personas como a sus allegados, al asegurar el cumplimiento de los deseos expresados previamente^1^, ^4^.

Uno de los principales retos en su aplicación es determinar el momento exacto en que una persona con demencia desea que no se prolongue su vida o que se considere la eutanasia. Para ello, se emplea la Escala de Deterioro Global (GDS) o Escala de Reisberg^5^, herramienta ampliamente utilizada para evaluar las etapas de deterioro cognitivo y facilitar decisiones acordes con la voluntad previamente manifestada.

En Lanzarote, la oficina de MAV inició su actividad en octubre de 2019 y en la actualidad cuenta con 2.144 registros activos. Está ubicada en el Hospital Universitario «Doctor José Molina Orosa» y ofrece atención tanto a personas hospitalizadas como a la población general. El servicio es gestionado por una enfermera con formación en bioética, responsable de asesorar sobre el procedimiento y tramitar el registro oficial. La citación se realiza preferentemente a través del servicio centralizado de atención telefónica (012).

Además de su función asistencial, la oficina desempeña un papel relevante en la promoción de los derechos sanitarios al final de la vida mediante charlas, talleres y campañas informativas dirigidas a profesionales y ciudadanía. Durante 2024, se llevó a cabo una recogida sistemática de datos con el fin de describir el perfil sociodemográfico de las personas que solicitaron el otorgamiento de un documento MAV y de analizar sus preferencias y nivel de satisfacción con el servicio.

El objetivo principal de este estudio fue describir las características sociodemográficas y los indicadores de preferencia en la realización del documento entre la población demandante de MAV en la isla de Lanzarote. Como objetivo secundario se analizó el grado de satisfacción de los usuarios con la atención recibida en la oficina de registro.

## Material y métodos

*Diseño del estudio.* Descriptivo de carácter prospectivo y corte longitudinal de un año de duración, en el cual se establecieron asociaciones con algunas variables sociodemográficas. Se realizó en el ámbito de la Atención Hospitalaria del Hospital Universitario «Doctor José Molina Orosa» de Lanzarote durante los meses de enero a diciembre de 2024.

La población de estudio fueron todos los usuarios que acudieron a la consulta de registro de MAV durante el periodo de estudio, que solicitaron al menos información, realizaron o modificaron el documento. Los criterios de inclusión incluían ser mayor de edad (18 años) y tener capacidad de obrar y actuar libremente. Se excluyeron a dos de los usuarios que no desearon participar en el estudio.

El instrumento empleado para la obtención de la información fue una bitácora de trabajo para las características sociodemográficas y los indicadores de preferencia, y un cuestionario de satisfacción en formato Likert (desde 1: totalmente en desacuerdo, hasta 5: totalmente de acuerdo) de elaboración propia *ad hoc.* Se recogieron variables sociodemográficas como la edad, el género, el país de nacimiento, el nivel de estudios y las preferencias religiosas. Además, se evaluaron las disposiciones en la cumplimentación del documento de MAV mediante variables como el motivo de realización, donación de órganos y tejidos o cuerpo para la ciencia, eutanasia, comunicación con familiares o seres queridos acerca del final de la vida, preferencias por el lugar para morir e instrucciones *post-mortem*, así como variables relacionadas con el lugar de registro, como la fuente de información, historial de contacto con la oficina y la relación con el ámbito sanitario. Para valorar la satisfacción se evaluó el proceso de citación, la información recibida y el trato proporcionado por el personal.

*Recogida de datos.* La información se recogió entre enero y diciembre de 2024, con la aprobación del Comité Ético de Investigación Clínica Provincial del Hospital Universitario «Doctor Negrín» (código 2023-366-1). Los usuarios firmaron el consentimiento informado antes de la entrevista, realizada durante la actividad asistencial habitual de asesoramiento. El sexo se registró según autodefinición como hombre o mujer. El cuestionario de satisfacción, de carácter voluntario, se entregó al finalizar la consulta.

Se realizó un análisis descriptivo de las características sociodemográficas, las preferencias y la satisfacción de los usuarios. Las variables categóricas se expresaron en frecuencias y porcentajes, y las numéricas como medias ± desviación estándar. Las comparaciones entre variables cualitativas se efectuaron mediante el test de chi-cuadrado, y entre variables cuantitativas mediante pruebas no paramétricas de Mann–Whitney o Kruskal–Wallis, tras verificar la distribución con Kolmogorov–Smirnov. Se consideraron diferencias significativas con *p* < 0,05. Los análisis se realizaron con SPSS v.29.0.1.0. (IBM SPSS Statistics 31.0.0.0., Gerencia de Servicios Sanitarios, Área de Salud de Lanzarote, Arrecife, Lanzarote, España)

## Resultados

Se analizaron 527 usuarios (edad media 59,5 ± 14,3 años; 67,2% mujeres), cuyas principales características y preferencias se recogen en las Tabla 1, Tabla 2.

**Tabla 1:** Perfil sociodemográfico de los usuarios que realizaron el documento de Instrucciones Previas (N = 527)

Variable	n (%) o media ± DE
*Edad (años)*	59,5 ± 14,3 (19-101)
*Sexo*	
Mujeres	354 (67,2)
Hombres	173 (32,8)

*País de nacimiento*	
España	379 (71,9)
Otros	148 (28,1)

*Nivel educativo*	
Sin estudios	12 (12,3)
Estudios primarios	114 (21,6)
Estudios secundarios	203 (38,5)
Estudios superiores	198 (37,6)

*Religión*	
No profesa	415 (78,7)
Católica	74 (14,0)
Testigo de Jehová	26 (4,9)
Otras (protestante, budista, etc.)	12 (2,3)

*Actividad profesional*	
Otros	417 (79,1)
Sanitarios/ trabajadores del ámbito asistencial	110 (20,9)

*Lugar de realización*	
Oficina habilitada	496 (94,1)
Planta de hospitalización	24 (4,6)
Domicilio	7 (1,3)

Nota metodológica: las variables cualitativas se expresan en frecuencia absoluta (n) y frecuencia relativa (%). Las variables cuantitativas se presentan como media ± desviación estándar (rango mínimo–máximo) sobre el total de casos válidos.

**Tabla 2:** Preferencias y actitudes de los usuarios ante la cumplimentación del documento de Instrucciones Previas a ver (N = 527)

Variable	n (%)
*Tipo de consulta*	
Primera consulta	322 (61,1)
Consulta sucesiva	205 (38,9)

*Motivo principal para realizar el documento*^a^	
Decidir por sí mismo	203 (38,5)
No ser una carga/ evitar sufrimiento	293 (55,6)
Motivos religiosos u otros	29 (5,5)

*Fuente de información sobre el documento*^b^	
Familiares o amigos	251 (47,6)
Medios, talleres o cartelería	170 (32,3)
Profesionales sanitarios/ jurídicos/ religiosos	80 (15,2)

*Decisiones relacionadas*	
Desea acogerse a la eutanasia	457 (86,7)
Desea donar órganos o tejidos	386 (73,2)
Desea donar el cuerpo a la ciencia	91 (17,3)

*Preferencias sobre el final de la vida*	
Prefiere incineración	424 (80,5)
Prefiere morir en domicilio	207 (39,3)
Sin preferencia	204 (38,7)

*Comunicación y actitud ante la muerte*	
No solicita información sanitaria	410 (77,8)
Habla abiertamente del tema con allegados	300 (56,9)
Considera que debe hablarse sin tabú	295 (56,0)

Nota metodológica:

a Este bloque integra las categorías decidir por sí mismo*,* no ser una carga*,* evitar sufrimiento*,* motivos religiosos y otras causas*,* agrupadas por afinidad conceptual.

b Incluye tanto fuentes informales de información (familiares, allegados, medios de comunicación, talleres o cartelería) como fuentes formales (profesionales sanitarios, jurídicos o religiosos). Las variables cualitativas se presentan en frecuencia absoluta (n) y frecuencia relativa (%) sobre el total de casos válidos. En aquellas variables que admiten más de una respuesta o proceden de preguntas analizadas de forma independiente, los porcentajes no son mutuamente excluyentes y pueden no sumar el 100%.

Entre los participantes que completaron la encuesta de satisfacción (n = 107; 20,3%), la mayoría gestionó su cita a través del teléfono de atención 012 (84,1%) y manifestó un alto grado de satisfacción con el proceso de citación (84,1%), el espacio físico (63,6%) y, especialmente, con la información y el trato recibidos (99,1%). Casi todos recomendarían la realización de la MAV a otras personas (99,1%).

En el análisis de asociaciones (tabla 3) se observaron diferencias significativas entre el nivel educativo y la solicitud de eutanasia (*p* < 0,001), así como entre el sexo y la religión respecto al motivo de realización del documento (*p* = 0,020 y *p* < 0,001, respectivamente). También se hallaron diferencias en la edad media según la actitud ante hablar sobre la muerte (*p* < 0,001), siendo los usuarios más jóvenes quienes consideraban necesario abordar el tema con naturalidad. No se encontraron diferencias significativas entre el sexo y las preferencias sobre el lugar de fallecimiento ni en el grado de demencia al que se acogerían a la eutanasia (*p* > 0,05).

**Tabla 3:** Análisis de asociaciones entre variables sociodemográficas y preferencias de los usuarios (N = 527)

Comparación	Categoría/grupo (variable explicativa)	Resultado (% o media ± DE	Estadístico	p-valor
**Nivel educativo y solicitud de eutanasia**	Sin estudios	50,0	χ^2^(6) = 39,27	< 0,001
	Primarios	79,6		
	Secundarios	85,1		
	Superiores	95,5		
**Sexo y motivo de realización**	Ser yo quien decida	Mujeres: 64,5 / Hombres: 35,5	χ^2^(4) = 11,70	0,020
	Motivos religiosos	Mujeres: 80,8 / Hombres: 19,2		
	No ser una carga	Mujeres: 74,3 / Hombres: 25,7		
	Evitar sufrimiento	Mujeres: 59,3 / Hombres: 40,7		
	Otras casusas	Mujeres: 33,3 / Hombres: 66,7		
**Religión y motivo de realización**	Ser yo quien decida	Ninguna: 43,3 / Católica: 24,3/ Testigos de Jehová: 3,8 / Protestante: 66,7 / Otros: 37,5	χ^2^(20) = 463,49	< 0,001
	Motivos religiosos	Ninguna: 0,0 / Católica: 2,7 / Testigos de Jehová: 92,3 / Protestante: 0,0 / Otros: 0,0		
	No ser una carga	Ninguna: 33,9 / Católica: 40,5 / Testigos de Jehová: 3,8 / Protestante: 33,3 / Otros: 25,0		
	Evitar sufrimiento	Ninguna: 22,5 / Católica: 29,7 / Testigos de Jehová: 0,0 / Protestante: 0,0 / Otros: 37,5		
	Otras casusas	Ninguna: 0,2 / Católica: 2,7 / Testigos de Jehová: 0,0 / Protestante: 0,0 / Otros: 0,0		
**Edad y actitud ante hablar sobre la muerte**	Familiares/allegados	62,6 ± 14,0	H(4) = 22,68	< 0,001
	Personal sanitario	66,0 ± 19,1		
	Ambos	56,6 ± 17,0		
	No se habla	67,2 ± 16,6		
	Con todos	57,7 ± 13,1		
**Sexo y GDS (grado de demencia al que se acoge la eutanasia)**	Sin deterioro/leve (GDS 0-3)	Hombres: 17,0 / Mujeres: 16,0	χ^2^(2) = 3,79	0,876
	Moderado (GDS 4-5)	Hombres: 63,9 / Mujeres: 60,6		
	Grave (GDS 6-7)	Hombres: 3,0 / Mujeres: 2,0		

Nota metodológica: Los porcentajes corresponden a la distribución dentro de cada categoría de la variable explicativa. En los contrastes entre variables cualitativas se aplicó la prueba de chi-cuadrado de Pearson (χ^2^), y en los casos que incluyeron variables numéricas no normales se empleó la prueba de Kruskal–Wallis (H). Los valores de *p* son bilaterales. En las variables multirrespuesta o procedentes de preguntas independientes, los porcentajes pueden no sumar el 100%.

## Discusión

Con respecto al perfil sociodemográfico, la mayoría de los usuarios que acudieron a la oficina eran mujeres (67,2%), con una edad media de 59 años, nacionalidad española y nivel educativo medio-alto. Este perfil coincide con lo descrito en otros estudios, donde se observa una mayor implicación femenina en la toma de decisiones anticipadas, atribuida a una mayor proactividad y planificación frente al final de la vida, mientras que los hombres tienden a delegar dichas decisiones en terceros^6^, ^7^.

La edad avanzada predominante concuerda con la literatura, que identifica una mayor predisposición en personas mayores, mientras que los grupos jóvenes perciben el documento como algo lejano o poco necesario^8^. Sin embargo, la presencia de un 7% de usuarios entre 19 y 35 años sugiere un incipiente cambio generacional, posiblemente vinculado a experiencias personales o familiares, que resalta la importancia de promover la reflexión anticipada sobre el final de la vida desde edades más tempranas.

En lo referente a las preferencias en la cumplimentación, el principal motivo para formalizar el documento fue el deseo de ejercer la autonomía personal (38,5%), en concordancia con estudios previos que señalan que las personas sin enfermedad activa son quienes más suelen planificar decisiones anticipadas^7^.

Las diferencias por confesión religiosa resultan notables: las personas aconfesionales priorizaron decidir por sí mismas (88,2%), los creyentes mencionaron no querer ser una carga (24,3%) y los Testigos de Jehová destacaron motivos religiosos (92,3%), en línea con la literatura^9^. Estos hallazgos refuerzan la necesidad de integrar las creencias personales y culturales en el proceso de decisión.

Casi la mitad de los usuarios conocieron la existencia del documento a través de familiares o amigos (47,6%), lo que coincide con otros estudios donde el entorno cercano actúa como principal fuente de información^10^. La limitada participación de los profesionales sanitarios como canal informativo (11%) pone de manifiesto una escasa implicación en la promoción del registro y refleja carencias formativas ya descritas^11^, ^12^. Este déficit podría explicar que, pese a que la mayoría de los usuarios considera necesario superar el tabú social sobre la muerte, muchos restringen la conversación al ámbito familiar.

Asimismo, el alto porcentaje de personas que no solicitaron información al personal sanitario sugiere una insuficiente sensibilización profesional y la necesidad de estrategias formativas específicas. La falta de capacitación limita la capacidad de asesorar y, por tanto, la efectividad del registro como herramienta de autonomía^11^.

En relación con las instrucciones *post mortem,* predomina la elección de la incineración (80,5%), tendencia que refleja la secularización progresiva de la sociedad y el distanciamiento de los ritos religiosos tradicionales. Un 38,7% no manifestó preferencia sobre el lugar de fallecimiento, lo que puede relacionarse con la dificultad de anticipar el proceso de morir o con una menor carga simbólica atribuida a este aspecto. El 86,7% de los usuarios se acogió a la posibilidad de recibir la eutanasia, especialmente quienes poseían estudios superiores, posiblemente por una mayor comprensión normativa y reflexión sobre la autonomía en el final de la vida. Los valores culturales y religiosos siguen siendo determinantes: tanto el cristianismo como el islam promueven la aceptación del sufrimiento como parte natural del proceso de morir^13^, ^14^, lo que podría explicar que un 12,9% de los usuarios no se pronunciara o rechazara esta opción.

La baja tasa de respuesta a la encuesta enviada por correo electrónico constituye una limitación del estudio, posiblemente relacionada con el carácter voluntario y la sensibilidad del tema. Aun así, los resultados aportan una visión útil de la percepción de los usuarios. También cabe considerar como limitación la posible existencia de sesgos de información, concretamente un sesgo de atención (efecto Hawthorne) y sesgo de obsequiosidad, derivados del uso de entrevistas estructuradas por la investigadora. Este último se intentó mitigar mediante la formulación neutral y homogénea de las preguntas.

El estudio se realizó en una única área de salud y durante un solo año, lo que limita la generalización de los resultados^15^. Aun así, los hallazgos aportan información valiosa y replicable en otras áreas sanitarias, contribuyendo al conocimiento sobre las características y percepciones de los usuarios del registro de instrucciones previas.

*Implicaciones en la práctica clínica y/o futuras líneas de investigación.* Los resultados permiten definir el perfil del usuario demandante y orientar estrategias de difusión y formación tanto a la población general como a los profesionales. Es necesario incluir la formación sobre las MAV y la LORE en los planes estratégicos de las direcciones sanitarias, de modo que los profesionales dispongan de competencias para abordar el final de la vida como parte de la atención integral.

Deben promoverse estrategias de comunicación proactivas en las que los sanitarios informen activamente sobre la MAV como medida preventiva, sin esperar a que el paciente lo solicite. El menor número de hombres que realizan el documento evidencia un sesgo de género^6^, ^7^, lo que exige acciones específicas para fomentar su participación.

Asimismo, conviene reforzar la captación de población joven mediante programas educativos en institutos y campañas que promuevan la cultura de la muerte digna como valor social. La creación de talleres, espacios de diálogo y materiales educativos puede contribuir a normalizar la conversación sobre el final de la vida en el entorno familiar.

La creciente demanda observada en Lanzarote requiere ampliar el número de profesionales de Atención Primaria acreditados para registrar las MAV, tal como ya ocurre en comunidades como Cataluña o el País Vasco^16^, ^17^. Es prioritario fomentar entre los profesionales sanitarios una cultura de respeto a la autonomía del paciente**,** facilitando el asesoramiento y el acceso al registro desde los centros de salud.

## Conclusiones

Los resultados de este estudio aportan una visión actualizada del perfil y las preferencias de las personas que formalizan el registro de instrucciones previas en un área insular, aportando evidencias comparables a las descritas en otros contextos del Sistema Nacional de Salud. El perfil mayoritario corresponde a una mujer española de 59 años, con estudios secundarios y sin adscripción religiosa, que realiza su MAV principalmente para no ser una carga y se acoge a la eutanasia. La satisfacción con el servicio es muy elevada. Las personas jóvenes muestran menor resistencia a hablar sobre la muerte, mientras que la mayoría de los usuarios opta por la incineración y no expresa preferencia por el lugar de fallecimiento.

Se requieren estrategias de difusión y captación dirigidas a colectivos menos representados (población masculina y con menor nivel educativo), así como programas de formación y sensibilización entre profesionales sanitarios y ciudadanía.

**Puntos clave**

**Lo conocido sobre el tema**

- El registro de Instrucciones Previas, regulado por la Ley 41/2002, garantiza la autonomía del paciente y en Canarias se denomina Manifestaciones Anticipadas de Voluntad (MAV).
- Su implantación es desigual entre comunidades autónomas y existe poca información publicada sobre su utilización real.
- La mayoría de los estudios previos abordan aspectos legales o la percepción de los profesionales, con escasa evidencia sobre los usuarios que formalizan el registro.

**¿Qué aporta este estudio?**

- Describe, por primera vez en el ámbito insular de Canarias el perfil sociodemográfico y las preferencias de las personas que registran una MAV.
- Identifica los motivos principales para realizar la declaración y el nivel de satisfacción percibido con la atención recibida.
- Aporta evidencia útil para orientar estrategias de información y sensibilización dirigidas a la ciudadanía y a los profesionales sanitarios sobre el ejercicio del derecho a decidir sobre los cuidados y tratamientos.

## Responsabilidades éticas

Las autoras declaran haber cumplido con la normativa vigente en materia de investigación y con los protocolos establecidos en su centro, contando con la aprobación del Comité Ético de Investigación Clínica Provincial del Hospital Universitario «Doctor Negrín» de Las Palmas (código 2023-366-1). Todos los participantes fueron informados y otorgaron consentimiento informado por escrito.

## Financiación

El trabajo no ha recibido financiación. La presente investigación no ha recibido ayudas específicas provenientes de agencias del sector público, sector comercial o entidades sin ánimo de lucro.

## Conflicto de intereses

Las autoras declaran no tener conflicto de intereses.
